# A Nightmare for Males? A Maternally Transmitted Male-Killing Bacterium and Strong Female Bias in a Green Lacewing Population

**DOI:** 10.1371/journal.pone.0155794

**Published:** 2016-06-15

**Authors:** Masayuki Hayashi, Masaya Watanabe, Fumiko Yukuhiro, Masashi Nomura, Daisuke Kageyama

**Affiliations:** 1 Graduate School of Horticulture, Chiba University, Matsudo, Chiba, Japan; 2 Insect Microbe Research Unit, National Institute of Agrobiological Sciences, 1–2 Owashi, Tsukuba, Ibaraki, Japan; International Atomic Energy Agency, AUSTRIA

## Abstract

For maternally transmitted microbes, a female-biased host sex ratio is of reproductive advantage. Here we found a strong female bias in a field population of the green lacewing, *Mallada desjardinsi* (Insecta; Neuroptera). This bias was attributed to the predominance of individuals harboring a maternally inherited male-killing bacterium that was phylogenetically closely related to the plant-pathogenic *Spiroplasma phoeniceum* and *Spiroplasma kunkelii*. Among 35 laboratory-reared broods produced by wild-caught females, 21 broods (60%)—all infected with *Spiroplasma*—consisted of only females (940 individuals). Among 14 broods consisting of both males and females (516 and 635 individuals, respectively), 4 broods were doubly infected with *Spiroplasma* and *Rickettsia*, 6 broods were singly infected with *Rickettsia*, and 3 broods were uninfected (remaining one brood was unknown). Mortality during embryonic and larval development was prominent in all-female broods but not in normal sex ratio broods. Following antibiotic treatment on all-female broods, mortality was significantly reduced and the sex ratio was restored to 1:1. Strong expression and high prevalence of this male-killer is remarkable considering its low density (~10^−5^–10^−4^ cells per host mitochondrial gene copy based on quantitative PCR). In addition, a bacterium closely related to *Rickettsia bellii* was present in 25 of 34 broods (73.5%), irrespective of the sex ratio, with the infection density comparable to other cases of endosymbiosis (~10^−2^–10^−1^ cells per mitochondrial gene copy). Higher density of *Rickettsia* than *Spiroplasma* was also demonstrated by electron microscopy which visualized both *Spiroplasma*-like cells and *Rickettsia*-like cells inside and outside the ovarian cells.

## Introduction

Unlike vertebrates, many invertebrates allow massive proliferation of certain groups of microbes, such as *Wolbachia*, *Rickettsia*, and *Spiroplasma*, within cytoplasm of their somatic and germ cells [1]. Because cytoplasmic elements are transmitted to offspring only through mothers, male hosts are non-profitable for such microbes.

Attention has been paid to some of these microbes because they can distort their host sex ratio toward female—the sex they are transmitted from—even at the expense of host fitness. Such sex ratio distortions, including male-killing, feminization, and parthenogenesis-induction, together with cytoplasmic incompatibility, are understood as selfish behaviors of microbes [2, 3].

Among these host manipulations, male-killing is induced by a wide diversity of microbes—a variety of bacteria such as *Spiroplasma*, *Wolbachia*, *Rickettsia*, and *Arsenophonus* [4]; microsporidian protists [5]; and a putative RNA virus [6]. Microbe-induced male-killing has previously been found from four insect orders (i.e., Diptera, Lepidoptera, Hymenoptera, and Coleoptera) and pseudoscorpions (Arachnida; Pseudoscorpionida). In most of these cases of male-killing, however, male-killers are harbored only by a small portion of individuals in a population, and therefore, ecological and evolutionary impact of male-killers are elusive (but see exceptions in male-killing *Wolbachia* in butterflies [7, 8]).

Here we report that a strong female-bias in a population of the lacewing *Mallada desjardinsi* (Neuroptera; Chrysopidae)—a species attracting attention because its larvae escape from the attack of aphid-tending ants by carrying aphid carcasses on their backs [9]—is caused by an endosymbiotic *Spiroplasma* bacteria. In addition, *M*. *desjardinsi* was found to be infected with *Rickettsia* bacterium that is not associated with sex ratio distortion. This is the first report of the occurrence of male-killing and the presence of *Rickettsia* and *Spiroplasma* in the insect order Neuroptera [10, 11], wherein XX/XY sex determination is considered to be common [12]. Lacewing larvae are voracious consumers of aphids and insect eggs and are used as a biological control agent in agriculture [13]. Our findings highlight the effects of male-killing *Spiroplasma* on sex ratios, and possibly on sexual behaviors and population demography of this agriculturally important insect.

## Materials and Methods

### Insects

Sixty-four adults of *M*. *desjardinsi* (Insecta; Neuroptera; Chrysopidae) were collected by a sweeping net under street lamps near trees and bushes in the campus of Chiba University, Matsudo, Chiba Pref., Japan, at night (20:00–22:00) from May to October in 2011. *M*. *desjardinsi* is not an endangered or protected species. Specific permission is not required for insect collection for students and faculty members in the campus of Chiba University. Females were brought into the laboratory and individually allowed to lay eggs in plastic containers for 15 days. During egg collection, females were fed with 50% honey solution and dried yeast. After egg collection, females were stored at −40°C until DNA extraction. To prevent cannibalism, an egg was placed in each well of the 24-well plate (cat. no., 142475, Nunc^™^ Cell-Culture Treated Multidishes, Thermo Fisher Scientific K.K., Yokohama, Japan) together with *Ephestia kuehniella* (Lepidoptera; Pyralidae; Agrisect Inc., Ibaraki, Japan) eggs, as larval diet. Insects were reared under a 16-h:8-h light:dark photoperiod at 25 ± 1°C. Adults were sexed according to the abdominal tip morphology.

### Antibiotic treatment

Six female adults of the line MK20 were fed with 50% honey solution containing tetracycline hydrochloride (0.1% w/w) for 7–8 days. The females were individually coupled with males and allowed to lay eggs for 1 month. Eggs laid by the females were reared as described above. As a control, we conducted the same procedure for six females of the same line using 50% honey solution that does not contain tetracycline hydrochloride.

### DNA extraction and diagnostic polymerase chain reaction (PCR)

DNA was extracted from adult abdomen using DNeasy^®^ Blood & Tissue Kit (Qiagen, Hilden, Germany). To assure DNA quality, partial sequence of mitochondrial cytochrome c oxidase subunit I (COI) gene was amplified by PCR using DNA barcoding primers (S1 Table). For the PCR detection of specific bacteria, partial sequence of *Spiroplasma spoT*, a gene sequence similar to that of the (p)ppGpp 39-pyrophosphohydrolase *spoT* gene, *Rickettsia* 16S ribosomal RNA (16S rRNA) gene, and *Wolbachia* surface protein (*wsp*) gene were amplified by respective primers (S1 Table). PCRs were conducted with Go-Taq^®^ (Promega KK, Tokyo, Japan).

### Quantification of bacterial densities by realtime PCR (qPCR)

Real-time fluorescence detection quantitative PCR (qPCR) was performed using SYBR Green and a LightCycler^®^ 480 System (Roche Diagnostics K.K., Tokyo, Japan). *Spiroplasma*-specific sequence was amplified using a primer pair, spoT-MDF designed for *M*. *desjardinsi Spiroplasma* (S1 Table) and spoT-r, *Rickettsia*-specific citrate synthase gene (*gltA*) sequence was amplified using glt375-F and glt574-R (S1 Table), and host mitochondrial COII sequence was amplified using C2-J3399 and C2-N3665 (S1 Table). For each of the reactions, standard samples, i.e., dilution series of PCR products (10^8^, 10^7^, 10^6^, 10^5^, 10^4^ and 10^3^ copies per microliter), were included in order to estimate the absolute copy numbers of the target sequence in the samples. To prepare standard samples, PCR products were gel-excised and purified by Wizard SV (Promega). Copy numbers of the standard samples were estimated by the concentration measured by a spectrophotometer, regarding that the molecular weight of a nucleotide as 309 g/mol. In each qPCR reaction, two replicates were performed with similar results. All qPCR reactions were performed using a temperature profile of 40 cycles of 95°C for 10 s, 57°C for 10 s, and 72°C for 10 s. The qPCR data were analyzed by the Absolute Quantification analysis using the Second Derivative Maximum method implemented in the LightCycler^®^ 480 Instrument Operator Software Version 1.5 (Roche).

### Cloning and sequencing

From the whole-insect DNA of *M*. *desjardinsi*, almost the entire length of the bacterial 16S rRNA gene (about 1.5 kb) was amplified by PCR with the primers 16SA1 and 16SB1 (S1 Table) under the temperature profile of 94°C for 10 min, followed by 35 cycles consisting of 94°C for 1 min, 55°C for 1 min, and 72°C for 1 min. The PCR product was electrophoresed in a 1.5% agarose gel, excised, purified by using a Wizard SV (Promega), and cloned with the TA cloning vector pGEM^®^-T Easy Vector Systems (Promega) and *Escherichia coli* DH5 competent cells (Takara Bio Inc., Kusatsu, Japan), in which ampicillin and 5-bromo-4-chloro-3-indolyl-beta-D-galactopyranoside (X-Gal) were used for the blue-white selection system. Products of colony PCR were filtered using S-300 (GE Healthcare Japan, Tokyo, Japan) and were subjected to sequencing reactions using BigDye Terminator v3.1 Cycle Sequencing Kit (Thermo Fisher Scientific) and the following sequencing primers: T7 and SP6 in the flanking regions of the vector and internal primers 16SA2 and 16SB2 (S1 Table). The sequencing products, filtered using Sephadex G-50 (GE Healthcare), were subjected to a DNA Analyzer (model 3730xl; Applied Biosystems). In addition, the RNA polymerase B (*rpoB*) gene amplified by the primers RpoBF1 and RpoBR4 (S1 Table) and (p)ppGpp 39-pyrophosphohydrolase *spoT* gene [14] amplified by the primers spoT-f and spoT-r (S1 Table) were gel-excised, purified, and directly sequenced.

### Transmission electron microscopy (TEM)

Ovaries of *M*. *desjardinsi* female adults were carefully dissected and excised with fine forceps under a stereo dissecting microscope. The ovaries were fixed in 2.5% glutaraldehyde (Nacalai Tesque, Kyoto, Japan) in 0.06 M phosphate buffer at 4°C for 2 h. After rinsing with the same buffer, the samples were postfixed with 2% osmium tetroxide at 4°C for 1 h and washed three times with 0.1 M sodium acetate for 10 min each. The samples were stained with 1% uranyl acetate for 20 min at room temperature and then sequentially dehydrated on ice twice with 70% ethanol for 5 min each and twice with 95% ethanol for 5 min each. The dehydrated samples were placed in Quetol 651 (Nisshin EM, Tokyo, Japan), a water-miscible resin, and embedded in a Quetol 651 resin mixture according to the manufacturer’s protocol. Ultrathin sections were stained with TI blue (Nisshin EM) and Sato’s lead solution, and observed under a JEM-1010 transmission electron microscope (JEOL, Tokyo, Japan).

### Phylogenetic analyses

To infer the phylogenetic position of the *Spiroplasma* among the other *Spiroplasma* species found to date, the nucleotide sequences of the 16S rRNA, *rpoB*, and *spoT* genes were used. Sequence alignments were performed using Clustal W [15] implemented in BioEdit [16]. The evolutionary history was inferred by using the Maximum Likelihood method based on the General Time Reversible model using MEGA6 [17]. Initial tree(s) for the heuristic search were obtained automatically by applying Neighbor-Join and BioNJ algorithms to a matrix of pairwise distances estimated using the Maximum Composite Likelihood (MCL) approach, and then selecting the topology with superior log likelihood value. A discrete Gamma distribution was used to model evolutionary rate differences among sites.

The nucleotide sequences of other *Spiroplasma* and *Rickettsia* were taken from the GenBank database (http://www.ncbi.nlm.nih.gov/).

### Statistical analyses

Survival data: Analyses on binary outcomes (i.e., alive or dead) were performed with generalized linear mixed models (GLMMs) with binomial error distribution to assess the effects of mothers on survival rates. Generalized linear mixed models are an extension to the generalized linear models [18] in which the linear predictor contains random effects in addition to the fixed effects [19]. To account for stochastic among-brood variation (i.e., repeated observation within single broods), we included the random effects of brood identity in the models. As some of the data sets contain hierarchical error structures (i.e., effects of mothers), they were included as the hierarchical random effects in the models. All these statistical analyses were calculated by the function *glmer* of the program package lme4 using the software R version 3.0.1 [20].

Bacterial density data: The qPCR data (either *Spiroplasma spoT* copies per COII copy or *Rickettsia gltA* copies per COII copy) were analyzed with generalized linear models using the software R version 3.0.1. Using the function *glm* originally implemented in R, we adopted a generalized linear model for Gaussian, inverse Gaussian, or gamma distributions, which was selected according to the Akaike information criterion. Multiple comparisons were performed with Bonferroni corrections.

## Results

### Female-biased sex ratio and the prevalence of *Spiroplasma* and *Rickettsia*

Of 64 wild-caught *M*. *desjardinsi* adults, 57 were female and 7 were male. Thirty-five females bore offspring and 34 females were subjected to diagnostic PCR after oviposition (DNA extracted from one female was lost). Among 34 females, 19 females were positive for both *Spiroplasma* and *Rickettsia* (S^+^R^+^), 6 females were positive only for *Spiroplasma* (S^+^R^−^), 6 females were positive only for *Rickettsia* (S^−^R^+^) and 3 females were negative for both *Spiroplasma* and *Rickettsia* (S^-^R^-^) (Table 1). None of the 34 females was positive for *Wolbachia*. Total number of laboratory-reared F_1_ offspring were 1575 females and 516 males (S2 Table), wherein the number of males was disproportionately higher compared to the sex ratio of wild-caught adults (*P* = 0.0111 by Fisher’s exact probability test), which may suggest, in this population, that males take less time for searching for females than in population with 1:1 sex ratio [7].

**Table 1:** Infection status of wild-caught *M*. *desjardinsi* females and the sex ratio of their offspring.

Infection status	Number of broods			
	All-female	Female-biased^a^	Normal^b^	Total
S^+^R^+^	15	2	2	19
S^+^R^−^	6	−	−	6
S^−^R^+^	−	1	5	6
S^−^R^−^	−	−	3	3

S^+^R^+^: doubly infected with *Spiroplasma* and *Rickettsia*.

S^+^R^−^: singly infected with *Spiroplasma*.

S^−^R^+^: singly infected with *Rickettsia*.

S^−^R^−^: non-infected.

^a^ Contain males but sex ratio significantly deviated from 1:1 (*P* < 0.05 by chi-squared test).

^b^ Sex ratio not significantly deviated from 1:1 (*P* > 0.05 by chi-squared test).

### *Spiroplasma* as a sex-ratio distorter

Among 35 broods produced by wild-caught females, 21 broods consisted of only females (n = 940), whereas 14 broods consisted of both males and females (516 and 635 individuals, respectively). All the 21 all-female-producing females were positive for *Spiroplasma* (Table 1; S2 Table). In contrast, *Rickettsia* was detected from 18 of 24 female-biased broods and 7 of 10 normal broods, suggesting that *Rickettsia* is irrelevant to the sex ratio distortion (Table 1; S2 Table). Moreover, cloning of the bacterial 16S rRNA gene from the all-female-producing female (#5) shows that all the 25 *E*. *coli* colonies contain a unique sequence of *Spiroplasma* (S4 Table), suggesting that other bacteria cannot be the cause of the all-female trait.

### Male-killing as the likely mechanism for all-female trait

**Fig 1:**
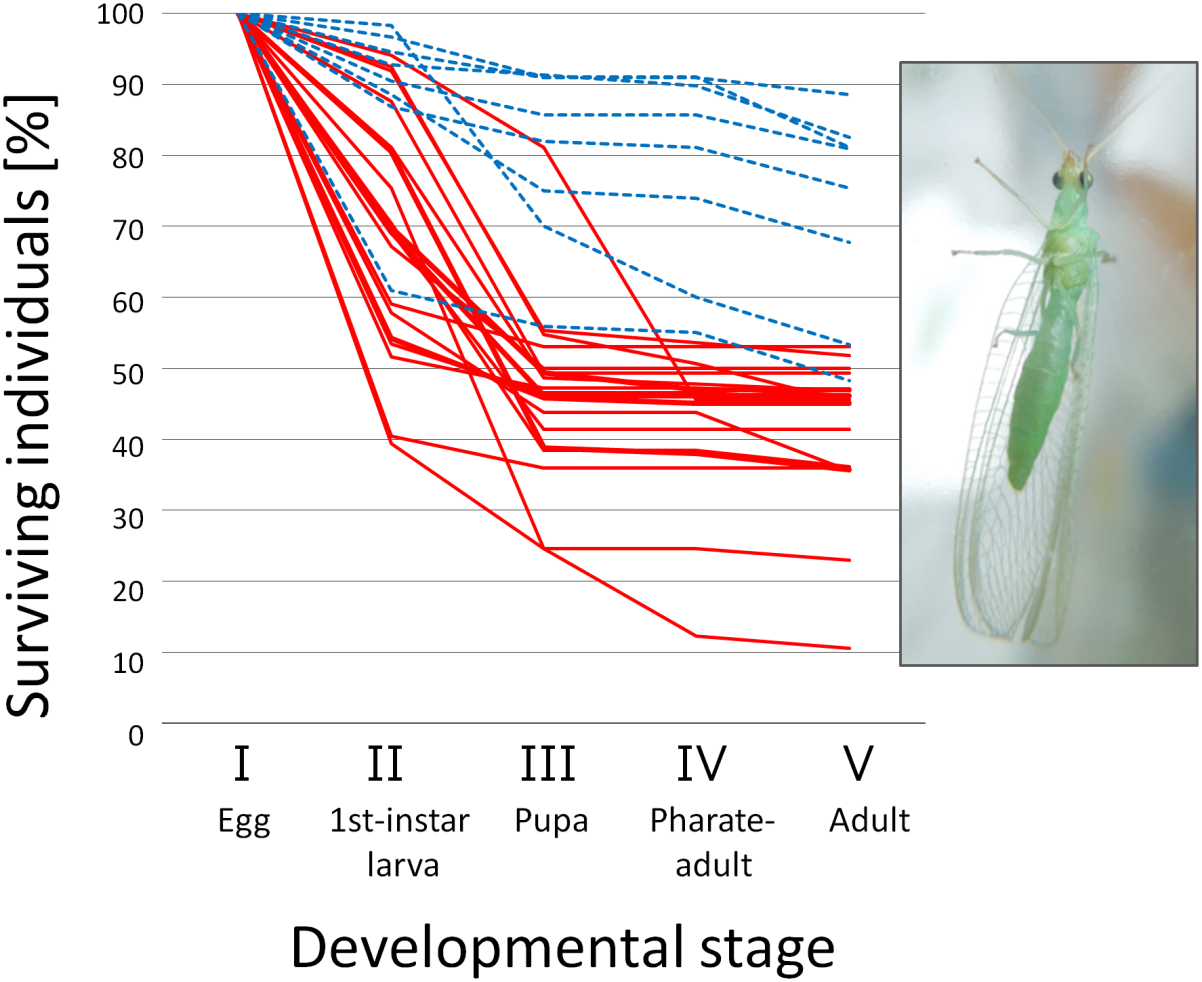
Survivorship of offspring produced by wild-caught females. Proportion of surviving individuals at the first-instar larval stage (II), the pupal stage (III), the pharate-adult (i.e., moving pupal) stage (IV), and the adult stage (V) among embryos (I). A solid line (red) represents a brood that developed to only females. A broken line (blue) represents a brood that developed to males and females with a ratio not significantly deviated from 1:1 (*P* > 0.05). A photograph of an adult female is given in the inset.

All-female broods suffered high mortality during embryonic and/or larval development, leading to nearly 50% or lower survival rate, whereas survival rates of normal sex ratio broods were higher than 50% except for one brood showing a survival rate of 48.3% (Fig 1). Survival rates of all-female broods were significantly lower than those of normal broods (*P* = 1.461 × 10^−7^).

Antibiotic treatment on females of the line MK20 during the adult stage significantly improved the survival rate during whole developmental stages (*P* = 0.0003), the egg hatch rate (*P* = 0.0003), but not the larval survival rate (*P* = 0.6921) of their offspring, resulting in nearly 1:1 sex ratio (Table 2). Diagnostic PCR showed that these offspring were free from *Spiroplasma*. Therefore, at least in MK20, male-killing during the embryonic stage is likely to be the mechanism for the all-female trait. Considering the variable timing of death in the progeny of the wild-caught females (Fig 1), timing of male-killing may not be restricted to the embryonic stage.

**Table 2:** Effects of tetracycline on survival rates and sex ratio of the matriline MK20.

Treatment	Brood	Eggs laid	No. of hatched larvae	No. adults			Proportion of females	Egg hatch rates^1^	Larval survival rates^2^	Survival rates^3^
				Female	Male	Total				
**(a) tetracycline-treated**										
	a	53	45	18	22	40	0.450	0.849	0.889	0.755
	b	58	57	23	29	52	0.442	0.983	0.912	0.897
	c	81	38	22	11	33	0.667	0.469	0.868	0.407
	d	52	44	19	19	38	0.500	0.846	0.864	0.731
	e	46	41	18	16	34	0.529	0.891	0.829	0.739
	f	80	72	33	35	68	0.485	0.900	0.944	0.850
**(b) non-treated**										
	g	28	11	7	0	7	1.000	0.393	0.636	0.250
	h	39	14	12	0	12	1.000	0.359	0.857	0.308
	i	121	61	52	0	52	1.000	0.504	0.852	0.430
	j	69	33	30	0	30	1.000	0.478	0.909	0.435
	k	166	76	70	0	70	1.000	0.458	0.921	0.422
	l	47	15	14	0	14	1.000	0.319	0.933	0.298

^1^No. of hatched larvae/ no. of eggs laid

^2^No. of adults/ no. of hatched larvae

^3^No. of adults/ no. of eggs laid

Despite the absence of *Spiroplasma*, a wild-caught female (#2) infected with *Rickettsia* produced progeny with a significantly female-biased sex ratio (70 females and 12 males; *P* = 1.286 × 10^−6^ by chi-squared test) (S2 and S4 Tables), possibly reflecting the sex-ratio-distorting ability of *Rickettsia*, which, however, remains elusive because this line was not maintained in the laboratory for further analyses (cf. *Rickettsia* was shown to be transmitted vertically to female-biased offspring; S5 Table). Low egg hatch rate (0.519) in brood #2 suggests that male-killing during embryogenesis might be the cause of the female-biased sex ratio (S2 Table).

### Localization and morphology of *Spiroplasma* and *Rickettsia*

**Fig 2:**
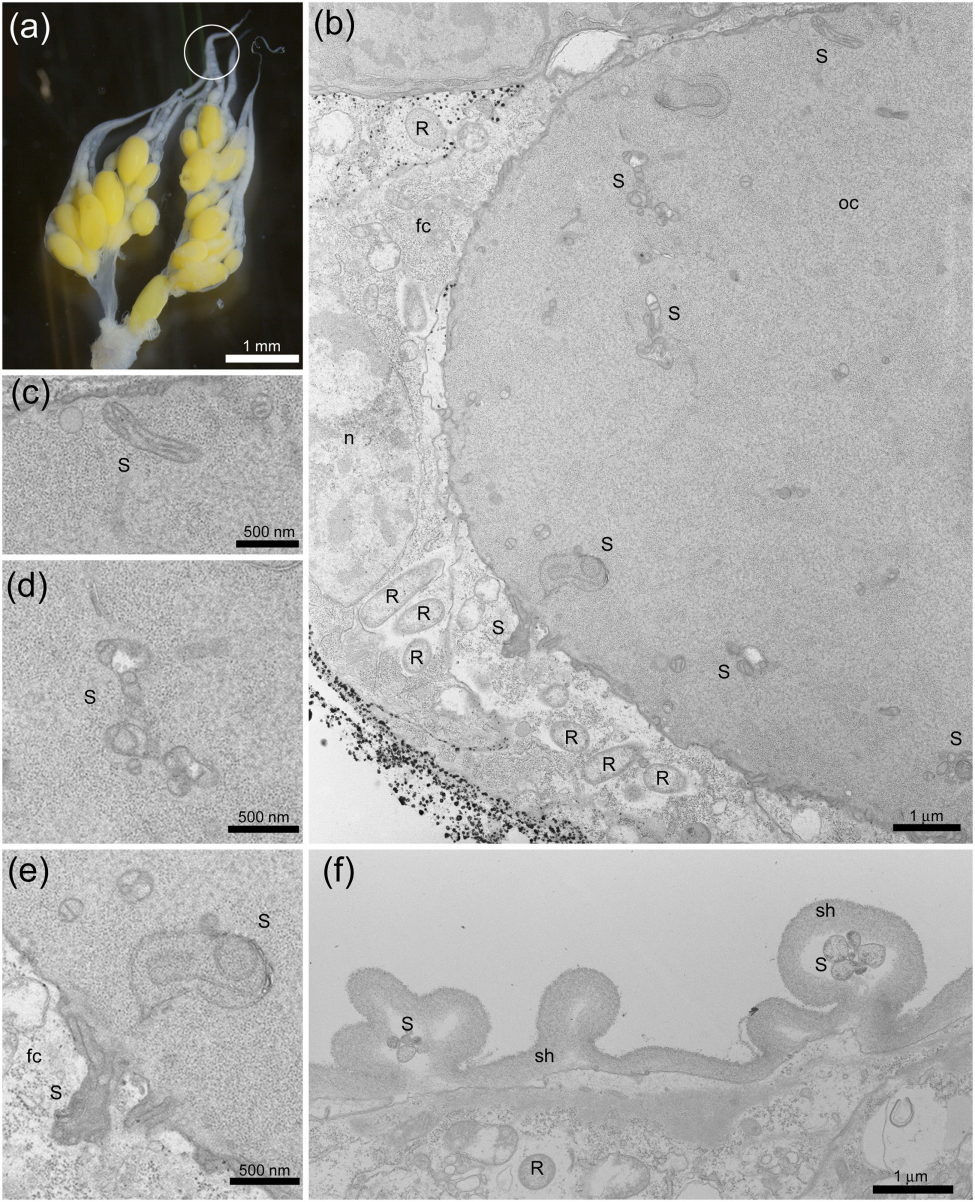
Transmission electron micrographs of *Spiroplasma* and *Rickettsia* in the female reproductive tissues of *M*. *desjardinsi*. **a**: Ovaries of a female *M*. *desjardinsi*. A circle represents a portion subjected to electron microscopy. **b**: Undifferentiated part of an ovariole, with *Rickettsia*-like structure in the follicle cells and *Spiroplasma*-like structure in the oocyte. **c**,**d, e**: Magnified images of (b), showing *Spiroplasma*-like structure. **f**: Differentiating part of an ovariole, with *Rickettsia*-like structure in the oocyte and *Spiroplasma*-like structure in the sheath. S: *Spiroplasma*; R: *Rickettsia*; oc: oocyte; fc: follicle cell; sh: sheath; n: nucleus.

**Fig 3:**
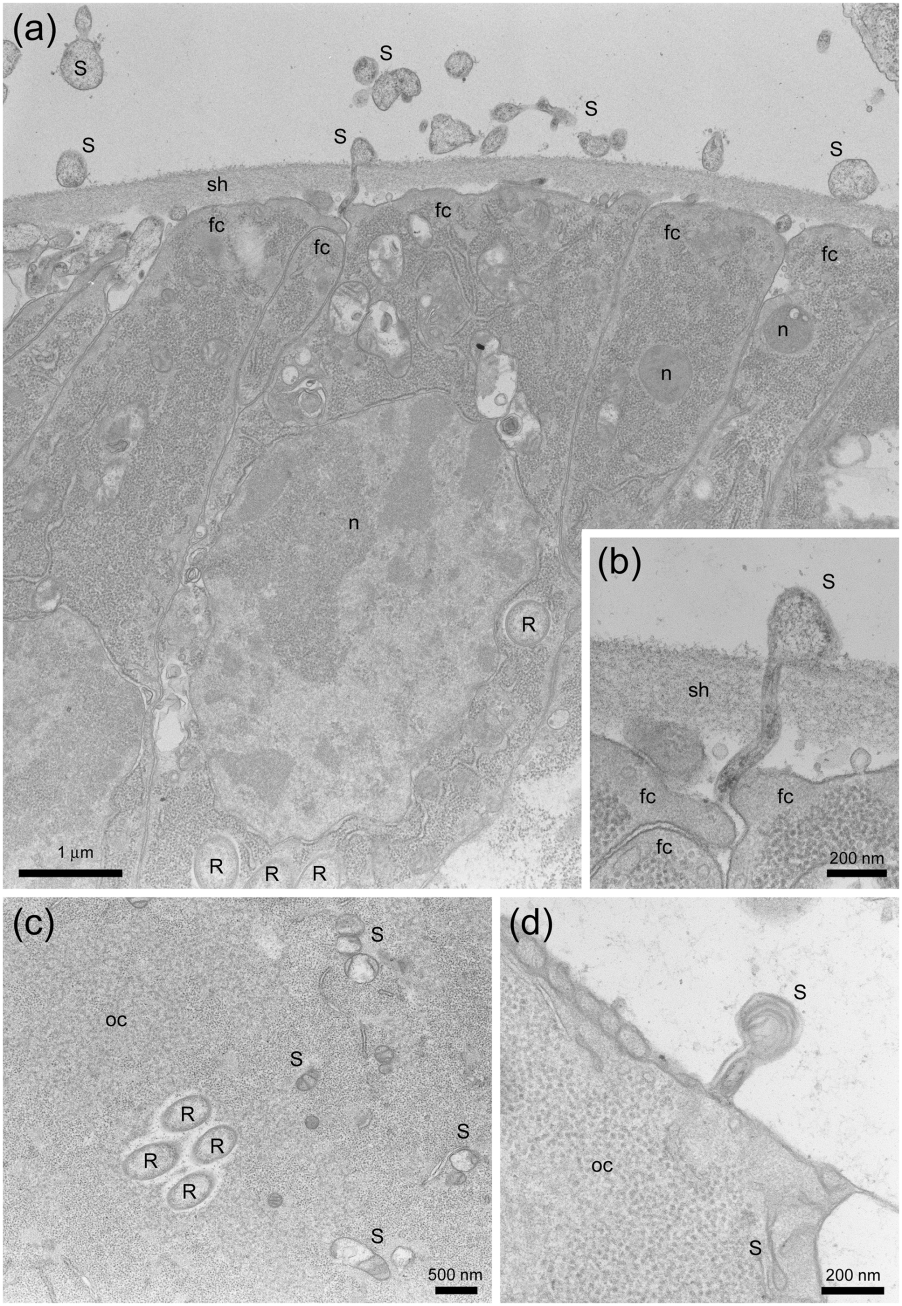
Further images of *Spiroplasma* and *Rickettsia* in the female reproductive tissues of *M*. *desjardinsi* observed by transmission electron microscopy. **a**: Differentiating part of an ovariole, with *Spiroplasma*-like structure outside and inside the sheath and *Rickettsia*-like structure in the follicle cells. **b**: Magnified image of (a), showing the presence of *Spiroplasma*-like structure in the intercellular space and the sheath, possibly at the timing of intrusion into a follicle cell. **c**: Cytoplasm of an oocyte, showing *Rickettsia* and *Spiroplasma*-like structure. **d**: *Spiroplasm*-like structure possibly at the timing of intrusion into the oocyte. S: *Spiroplasma*; R: *Rickettsia*; oc: oocyte; fc: follicle cell; sh: sheath; n: nucleus.

In undifferentiated parts of ovarioles (i.e., germarium) (located at the upper end within the circle in Fig 2a), a round-shaped *Rickettsia*-like structure [21] was abundantly observed in the follicle cells but no such structure was observed in oocytes (Fig 2b). In well-differentiated parts of ovarioles (located at the bottom end within the circle in Fig 2a), however, the *Rickettsia*-like structure was also observed in oocytes (Fig 3c), as well as in follicle cells (Fig 2f) and nurse cells (Fig 3a), possibly suggesting that *Rickettsia* intrude into the cytoplasm during egg maturation, which has been demonstrated in *Spiroplasma poulsonii* infecting *Drosophila melanogaster* [22].

Although much less in frequency, *Spiroplasma*-like pleomorphic structure [10, 23]—often recognized as twisted and half collapsed—was present in the oocytes (Fig 2b, 2c, 2d and 2e) in the undifferentiated parts of ovarioles. The *Spiroplasma*-like structure was also observed in the sheath cells (Figs 2f, 3a and 3b) and nurse cells (Fig 3a), as well as in the intercellular space (Fig 3b and 3d).

### Phylogenetic position of *Spiroplasma* and *Rickettsia*

**Fig 4:**
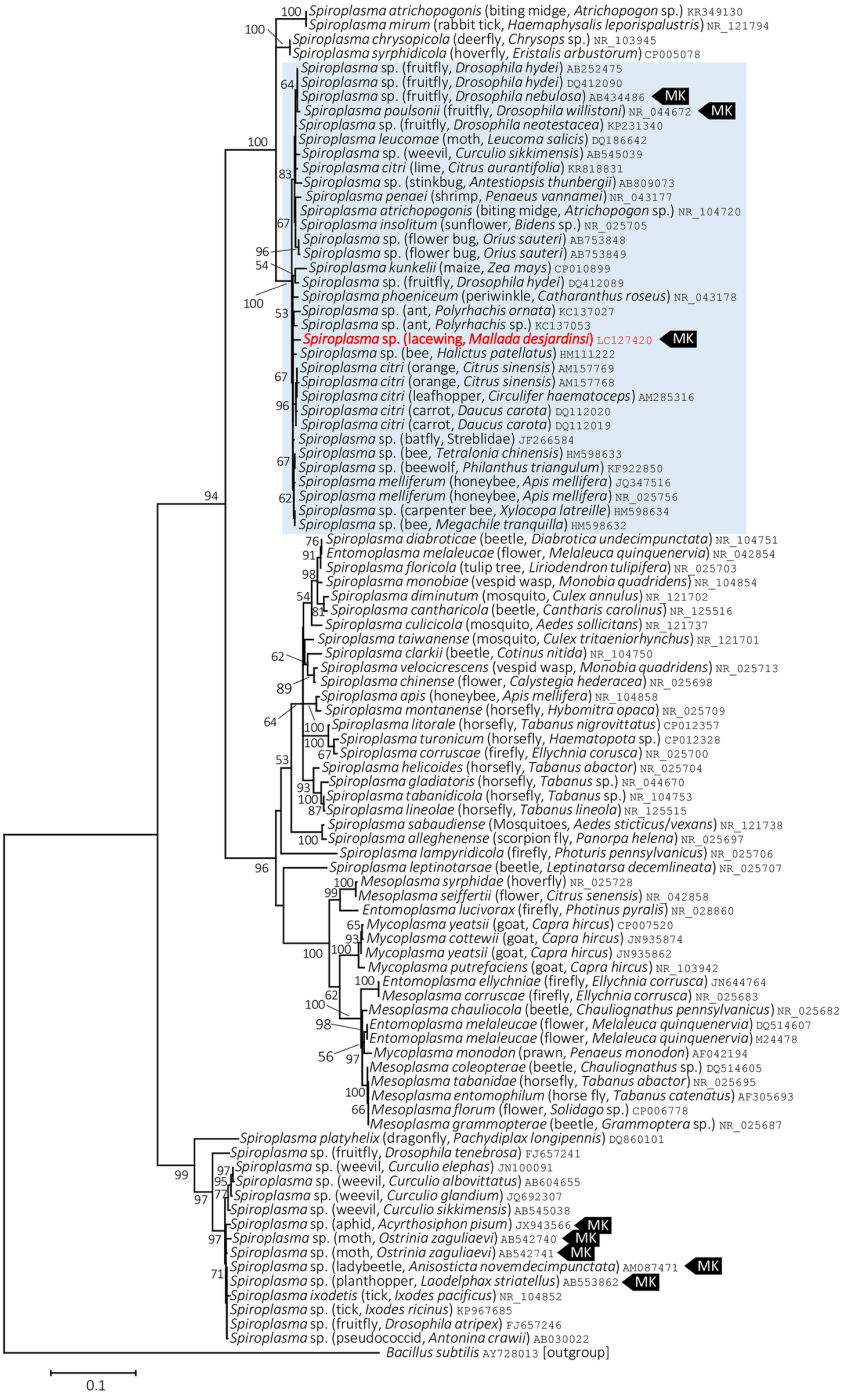
Phylogenetic position of *M*. *desjardinsi Spiroplasma* among *Spiroplasma* bacteria and relatives based on 16S rRNA gene sequences. A maximum likelihood tree (lnL = −6745.9487) is shown. Bootstrap probabilities are given at the nodes. *M*. *desjardinsi Spiroplasma* is depicted as red. The scale bar indicates the number of nucleotide substitutions per site. A clade containing *S*. *poulsonii*, *S*. *insolitum*, *S*. *kunkelii*, *S*. *phoeniceum*, *S*. *citri* and *S*. *melliferum* is shaded with light blue. A host organism is given in parenthesis. An accession number is given at the end of each OTU. MK represents a male-killer.

**Fig 5:**
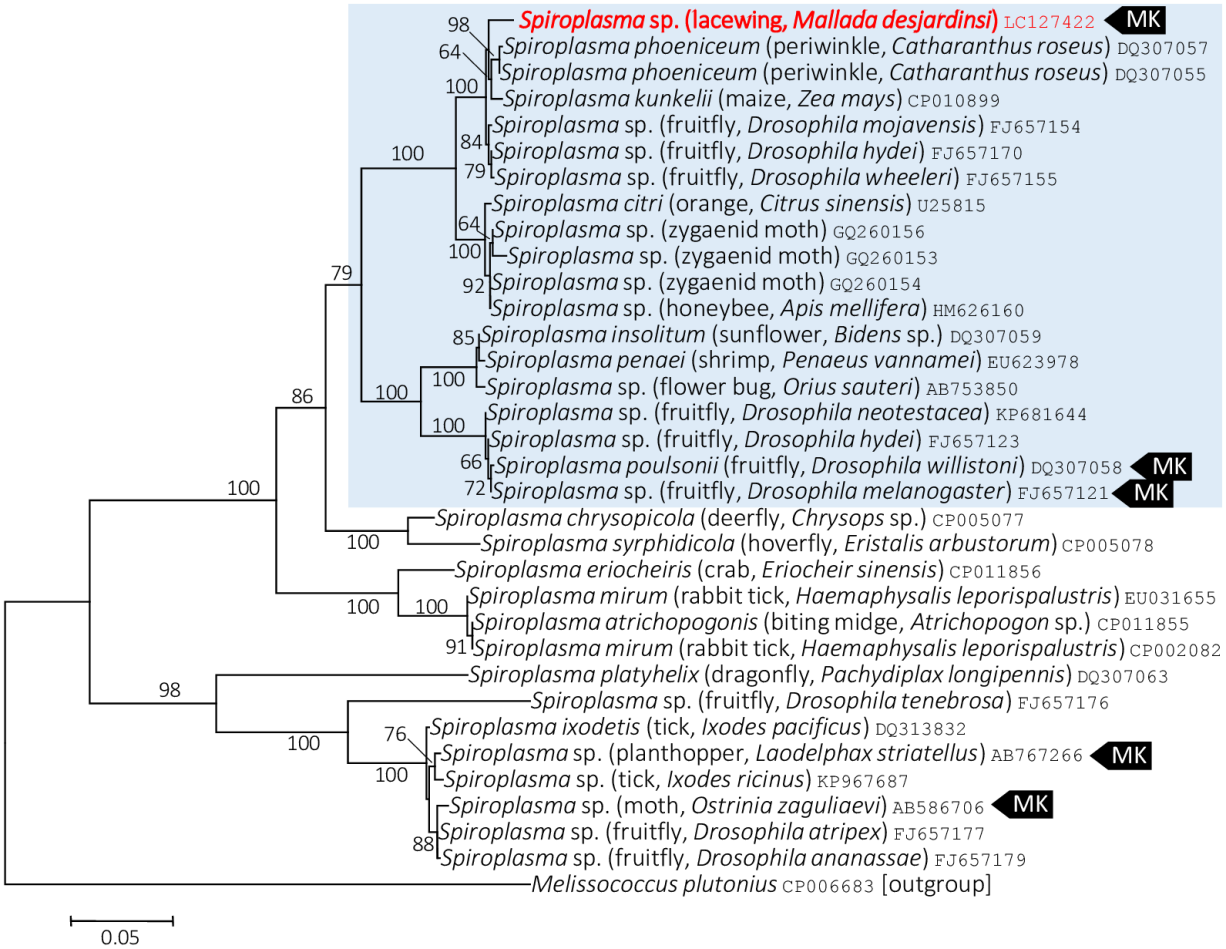
Phylogenetic position of *M*. *desjardinsi Spiroplasma* among *Spiroplasma* bacteria and relatives based on *rpoB* gene sequences. A maximum likelihood tree (lnL = −5057.9807) is shown. Bootstrap probabilities are given at the nodes. *M*. *desjardinsi Spiroplasma* is depicted as red. The scale bar indicates the number of nucleotide substitutions per site. A clade containing *S*. *poulsonii*, *S*. *insolitum*, *S*. *kunkelii*, *S*. *phoeniceum*, *S*. *citri* and *S*. *melliferum* is shaded with light blue. A host organism is given in parenthesis. An accession number is given at the end of each OTU. MK represents a male-killer.

**Fig 6:**
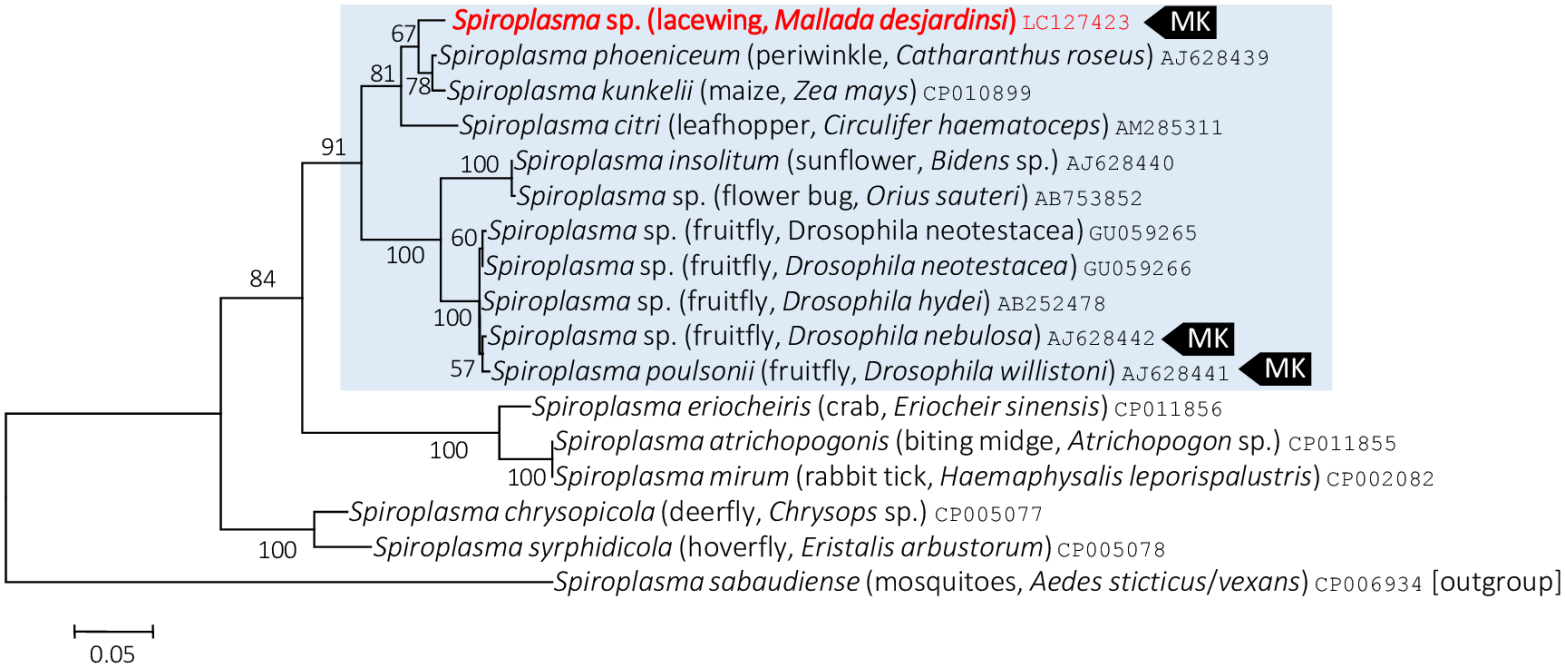
Phylogenetic position of *M*. *desjardinsi Spiroplasma* among *Spiroplasma* bacteria and relatives based on spot gene sequences. A maximum likelihood tree (lnL = −2525.6794) is shown. Bootstrap probabilities are given at the nodes. *M*. *desjardinsi Spiroplasma* is depicted as red. The scale bar indicates the number of nucleotide substitutions per site. A clade containing *S*. *poulsonii*, *S*. *insolitum*, *S*. *kunkelii*, *S*. *phoeniceum*, *S*. *citri* and *S*. *melliferum* is shaded with light blue. A host organism is given in parenthesis. An accession number is given at the end of each OTU. MK represents a male-killer.

On the Maximum Likelihood tree based on the 16S rRNA gene sequences, *Spiroplasma* present in *M*. *desjardinsi* falls in the Citri-Poulsonii clade (Fig 4), a large group consisting of *S*. *citri*, *S*. *poulsonii*, *S*. *phoeniceum*, *S*. *kunkelii*, *S*. *insolitum*, as well as *S*. *melliferum*. Within this large clade, *M*. *desjardinsi Spiroplasma* was placed outside the clade consisting *S*. *poulsonii* and *S*. *insolitum*, but the relationship with other *Spiroplasma* members was unclear (Fig 4; S1 Fig). However, Maximum Likelihood tress based on *rpoB* and *spoT* gene sequences gave a better resolution—they consistently showed that *Spiroplasma* present in *M*. *desjardinsi* is closely related to *S*. *phoeniceum* and *S*. *kunkelii* (Figs 5 and 6). The same results were obtained by Neighbor Joining and Maximum Parsimony methods.

**Fig 7:**
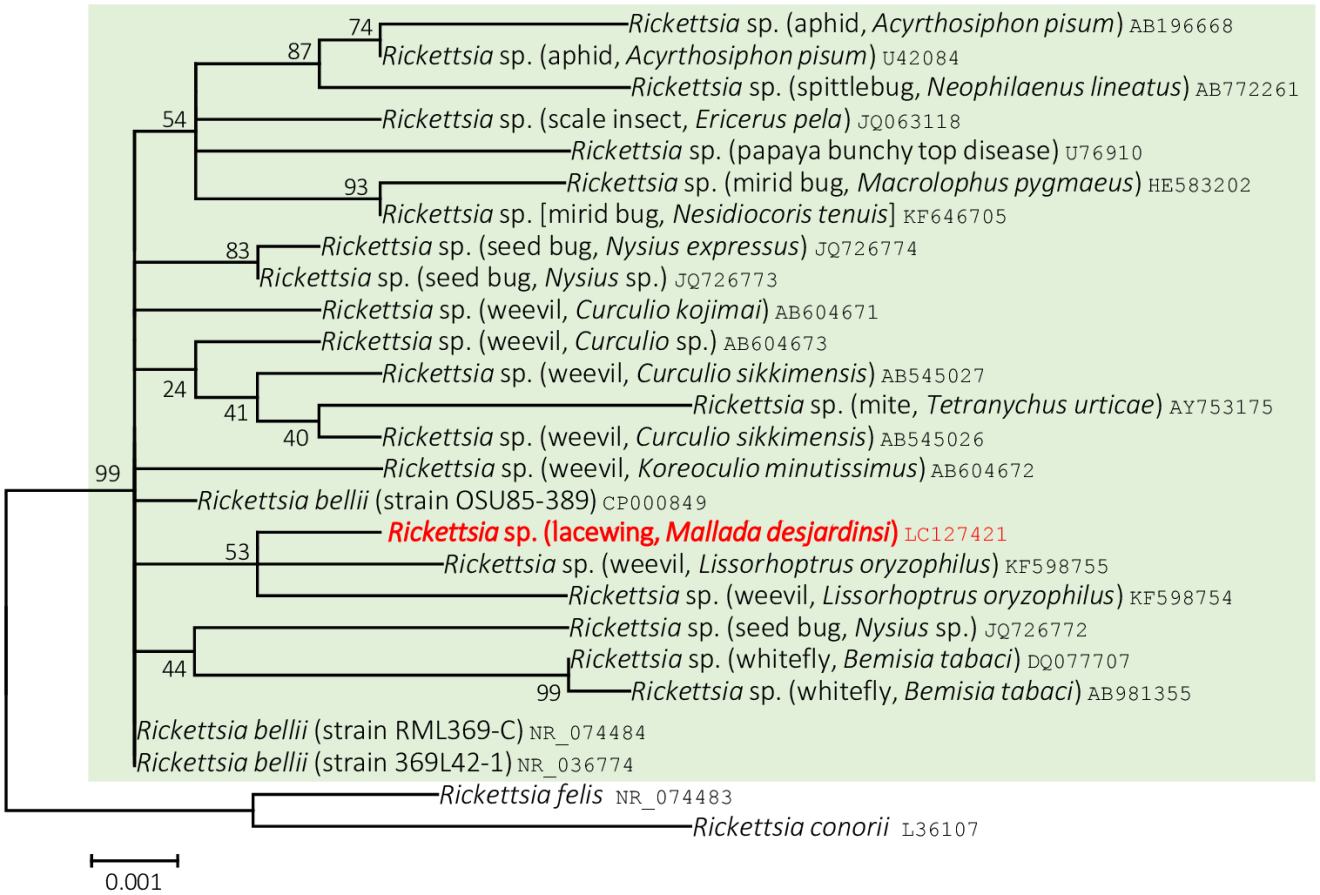
Phylogenetic position of *M*. *desjardinsi Rickettsia* among *R*. *bellii* relatives. A maximum likelihood tree (lnL = −2633.5879) based on 16S rRNA gene sequences. Bootstrap probability is shown next to the branches. *M*. *desjardinsi Rickettsia* is depicted as red. The scale bar (0.001) indicates the number of nucleotide substitutions per site. *R*. *bellii* and its relatives are shaded with light green. A host organism is given in parenthesis. An accession number is given at the end of each OTU.

Maximum Likelihood tree based on 16S rRNA gene clearly showed that *Rickettsia* present in *M*. *desjardinsi* is a member of *Rickettsia bellii* relatives (Fig 7), which was also supported by Neighbor Joining and Maximum Parsimony methods.

### Densities of *Spiroplasma* and *Rickettsia*

**Fig 8:**
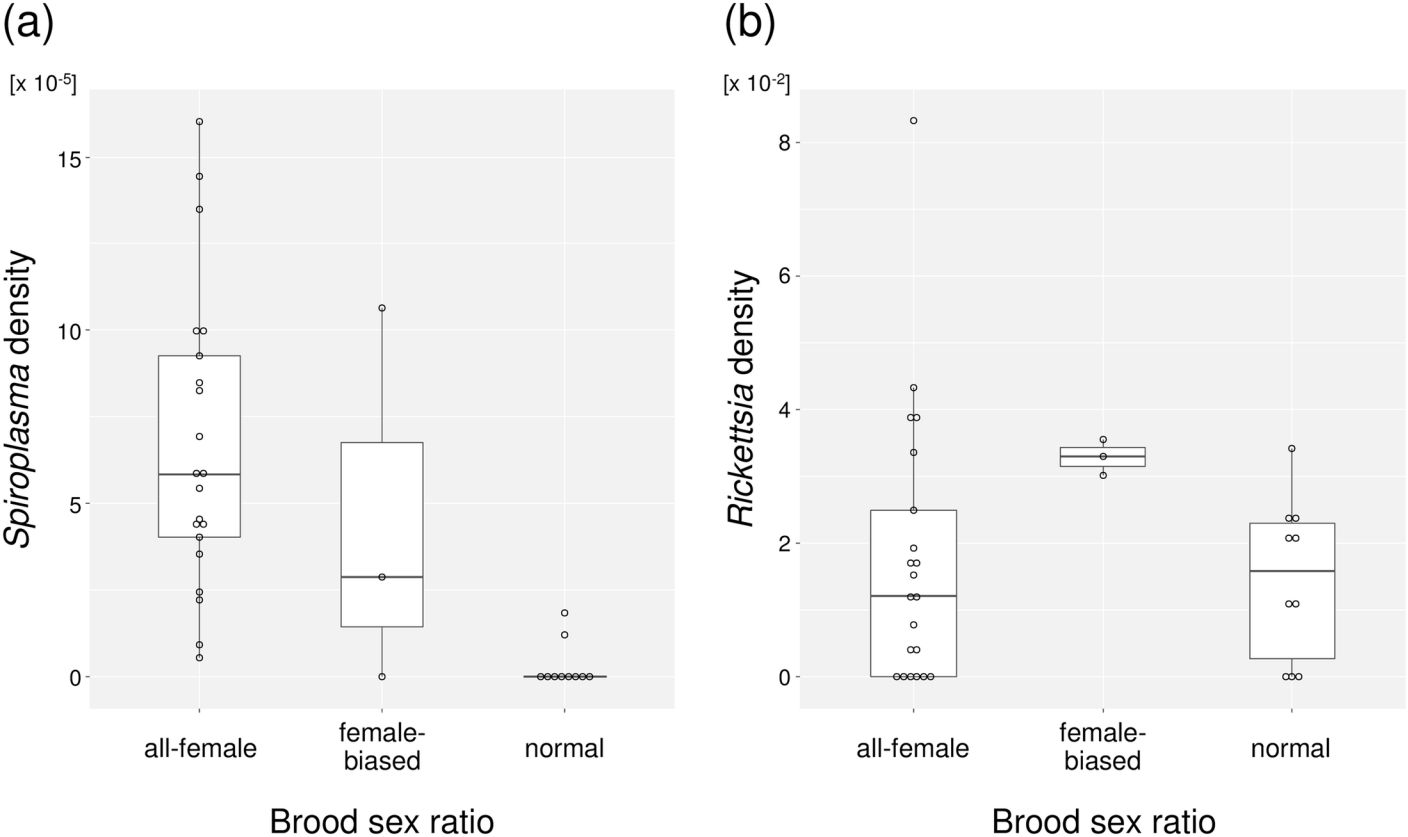
qPCR-estimates of endosymbiont population densities in mothers that are categorized by their offspring sex ratio. (a) *Spiroplasma* density in mothers (i.e., Relative values of *Spiroplasma spoT* gene copies per mitochondrial COII gene copy). (b) *Rickettsia* density in mothers (i.e., Relative values of *Rickettsia gltA* gene copies per mitochondrial COII gene copy).

According to the qPCR data (S3 Table), although two normal-sex-ratio-producing females (#21 and #46) showed significantly smaller *Spiroplasma* density than all-female-producing females (*P* = 0.0087), they also showed higher *Spiroplasma* density than two of the all-female-producing females (#10 and #20), which showed lowest *Spiroplasma* densities (Fig 8a). Hence, the density threshold may not explain the lack of male-killing expression in the two broods. Rather, suppressors against male-killing might exist in the genetic background of *M*. *desjardinsi*. In contrast, qPCR data showed that *Rickettsia* densities were not significantly different between mothers producing all-female and normal sex-ratio progeny (Fig 8b; *P* = 0.6059), supporting the view that the presence of *Rickettsia* was irrelevant to the host sex ratio.

Infection status examined for some of the F_1_ offspring showed that both *Spiroplasma* and *Rickettsia* were stably inherited in all-female broods (S5 Table). Notably, in a female-biased brood (#9), all the examined females (n = 12) were positive for both *Spiroplasma* and *Rickettsia*, while all the males (n = 7) were positive only for *Rickettsia* (S5 Table). Failure of vertical transmission of *Spiroplasma* may be the cause for the rescue of males in this brood. However, another female-biased brood produced by a doubly infected mother (#30) did not show such pattern; among 12 females and 12 males examined, all but one female were doubly infected with *Spiroplasma* and *Rickettsia* (S5 Table).

## Discussion

This study revealed that the frequent occurrence in *M*. *desjardinsi* of male-killing is the likely cause of female-biased population sex ratio of this species. The male-killing in *M*. *desjardinsi* is most likely caused by a *Spiroplasma* endosymbiont. According to previous reports, infection frequencies of male-killing *Spiroplasma* among host populations are variable; 30.77% in a ladybird beetle *Anisosticta novemdecimpunctata* [24], 0%–70% in another ladybird beetle *Adalia bipunctata* [25], 2%–3% in *Drosophila* flies [10, 26], 40% in a butterfly *Danaus chrysippus* [27], and only one instance in a moth *Ostrinia zaguliaevi* [28]. Considering the markedly high male-killing frequency (61.76%), the *Spiroplasma*–*M*. *desjardinsi* relationship would be an interesting biological system to investigate the evolutionary forces acting on insect sex ratio. Under such a highly female-biased condition, suppressors against male-killing and/or male-biased sex-ratio distorters would be expected to spread when they arise. The disproportionately female-biased sex ratio in wild-caught individuals in comparison with the breeding data may suggest the alteration of sexual behavior—males may spend less time for searching females, and hence, be less conspicuous—under the highly female-biased condition in *M*. *desjardinsi*. Moreover, genetic alterations related to sexual traits may be caused by persistence of female-biased sex ratio [29]. Alteration of sexual behaviors due to the *Wolbachia*-mediated scarcity of males have been suggested for a butterfly [7].

On the basis of molecular phylogenetic analyses, *Spiroplasma* found in the present study—a male-killer of *M*. *desjardinsi*—was unexpectedly closely related to *S*. *phoeniceum* and *S*. *kunkelii*. Both *S*. *phoeniceum* and *S*. *kunkelii* are plant pathogens, causing periwinkle yellowing disease and corn stunt disease, respectively [30, 31], and are closely related to *S*. *citri*, the causal agent of citrus stubborn disease. Although *S*. *citri* and *S*. *kunkelii* are known to be transmitted by leafhoppers, wherein spiroplasmas can proliferate in the hemolymph [32], it is unknown whether these spiroplasmas can be vertically (i.e., cytoplasmically) transmitted from mothers to offspring in the leafhoppers. Then, why is *Spiroplasma* in *M*. *desjardinsi* closely related to the plant pathogens? Although *M*. *desjardinsi* is a predatory insect and feeds on small insects in larval stages, it feeds on flower nectar in adult stage [13]. We consider that the common ancestors of *M*. *desjardinsi Spiroplasma* and its closely related plant pathogenic spiroplasmas might be at the interface between horizontal and vertical transmission of insect endosymbionts, and between plant pathogenicity and insect pathogenicity (i.e., male-killing). Further exploration of other spiroplasmas may lead to a better understanding of the evolution of these distinct phenotypic traits of spiroplasmas.

All the male-killing *Spiroplasma* discovered to date fall into *S*. *ixodetis* clade (moth, butterfly, and ladybird beetle) or *S*. *poulsonii* clade (*Drosophila*). Therefore, our finding of male-killing *Spiroplasma* in a new clade is suggestive of the possible occurrence of male-killers also from other *Spiroplasma* clades.

The timing of male-killing in *M*. *desjardinsi* was not restricted to the embryonic stage but was extended to later larval stages, suggesting that it may not be appropriate to be called early male-killing—the most common form of male-killing [33]. Such ambiguous timing of male-killing has also been reported in the *Wolbachia*-infected moths of the genus *Ostrinia* [34, 35], while timing of male-killing is restricted to embryonic stage in *Spiroplasma*-infected *Ostrinia zaguliaevi* [28]. Kageyama et al. [36] demonstrated, in *Spiroplasma*-infected *Drosophila melanogaster*, that the timing of male-killing is dependent on maternal age, and hence *Spiroplasma* titer. In *M*. *desjardinsi*, however, the timing of male-killing does not seem to be attributed to the *Spiroplasma* titers in mothers (S3 Table).

The low titer of *Spiroplasma* in *M*. *desjardinsi* revealed in the present study is reminiscent of the sex-ratio-distorting *Wolbachia* (strain *w*Fem) in the butterfly *Eurema mandarina* [37], which shows 10^−6^–10^−9^ copies of *Wolbachia wsp* gene per mitochondrial COI copy [38]. These results may suggest that a small amount of bacteria-derived products are sufficient to manipulate insect sex ratios. Low density within insect cells may render the *Spiroplasma* prone to fail in vertical transmission, where a strong bottleneck is assumed. This drawback might be counter-balanced by the reproductive advantage through male-killing [4, 33] or other possible effects on the host [39].

The 1:1 sex ratio exhibited in the broods produced by two of the *Spiroplasma*-positive females was not likely to be attributed to the *Spiroplasma* titers. Considering the fact that nucleotide sequences of the three genes (16S rRNA, *spoT* and *RpoB*) were indistinguishable between *Spiroplasma* in females producing all-female and 1:1 sex ratio progeny, we suspect the presence of host suppressors against male-killing as has been reported in a butterfly *Hypolymnas bolina* [40], which, however, remains unproven by genetic analyses in *M*. *desjardinsi*. At present, therefore, some mutations in the *Spiroplasma* genome cannot be excluded as the cause of the 1:1 sex ratio.

High prevalence of *Rickettsia* infection in *M*. *desjardinsi* is also intriguing. Although no apparent effects on host fitness were detected under laboratory condition in the present study, some unknown context-dependent effects on hosts [39, 41] might be attributed to the maintenance of this bacterium in the *M*. *desjardinsi* population.

In sum, a maternally transmitted male-killing *Spiroplasma* is the main cause of female-biased sex ratio observed in *M*. *desjardinsi*. Close relatedness of this *Spiroplasma* to plant-pathogenetic *S*. *phoeniceium*, *S*. *kunkelii* and *S*. *citri* based on molecular phylogenetic analyses may suggest that *M*. *desjardinsi Spiroplasma* is at the interface between horizontal and vertical transmission of insect endosymbionts, and between plant pathogenicity and insect pathogenicity (i.e., male-killing). Strikingly low density of *Spiroplasma* (10^−5^–10^−4^ cells per host mitochondrial gene copy) in *M*. *desjardinsi* is suggestive when considering the evolution of endosymbiosis and host manipulation. Presence of some individuals that escape male-killing may suggest the existence of suppressors against male-killing, which should be examined in future studies. In addition, a portion of *M*. *desjardinsi* individuals harbor *Rickettsia* endosymbiont which seems to have no effects on host sex ratio.

## Supporting Information

S1 Fig Topology of Maximum Likelihood tree of *Spiroplasma* based on 16S rRNA gene with bootstrap values on its nodes. (TIF)

S1 Table PCR primers used in this study. (PDF)

S2 Table Survivorship and sex ratio of F_1_ offspring. (PDF)

S3 Table qPCR data performed for wild-caught females, which were DNA extracted posterior to oviposition. (PDF)

S4 Table Bacterial contents inferred from the 16S rRNA gene sequencing. (PDF)

S5 Table Infection status of F_1_ offspring. (PDF)
