# Supplementary material for: A Nightmare for Males? A Maternally Transmitted Male-Killing Bacterium and Strong Female Bias in a Green Lacewing Population
Source: PLoS One. 2016 Jun 15;11(6):e0155794. doi: 10.1371/journal.pone.0155794 (PMC4909225; doi:10.1371/journal.pone.0155794)
Supplement: S1 Table — (PDF) [file pone.0155794.s002.pdf]

S1 Table. PCR primers used in this study.

Primers	Sequence (5' -> 3')	Target	Amplicon length (bp)	References
spoT-f (sense)	CAAACAAAAGGACAAATTGAAG	Spiroplasma spoT	513 (spoT-f & spoT-r)<br>222 (spoT-MDF & spoT-r)	Montenegro et al. (2005)
spoT-r (antisense)	CACTGAAGCGTTTAAATGAC			
spoT-MDF (sense)	TGCCAATTCCATATGAAGAC			this study
RpoBF1 (sense)	ATGGATCAAACAAATCCATTAGCAGA	Spiroplasma RpoB	1292	Haselkorn et al. (2009)
RpoBR4 (antisense)	CTTTGTTTCCATGGCGTCCAGCC			
wsp81F (sense)	TGGTCCAATAAGTGATGAAGAAAC	Wolbachia wsp	611	Braig et al. (1998)
wsp691R (antisense)	AAAAATTAAACGCTACTCCA			
Rb-F (sense)	GCTCAGAACGAACGCTATC	Rickettsia 16S rRNA	~900	Gottlieb et al. (2006)
Rb-R (antisense)	GAAGGAAAGCATCTCTGC			
glt375-F (sense)	TGGTATTGCATCGCTTTGGG	Rickettsia gltA	200	Caspi-Fluger et al. (2011)
glt574-R (antisense)	TTTCTTTAAGCACTGCAGCACG			
16SA1 (sense)	AGAGTTTGATCMTGGCTCAG	bacterial 16S rRNA	~1500 (16SA1 & 16SB1)	Fukatsu and Nikoh (1998)
16SB1 (antisense)	TACGGYTACCTTGTTACGACTT			
16SA2 (sense)	GTGCCAGCAGCCGCGGTAATAC			
16SB2 (antisense)	CGAGCTGACGACARCCATGCA			
C2-J3399 (sense)	ACAATTGGTCAYCAATGATAYTG	mitochondrial COII	267	Simon et al. (2006)
C2-N3665 (antisense)	CCACAAATTTCTGAACATTG			
LCO1490 (sense)	GGTCAACAAATCATAAAGATATTGG	mitochondrial COI	709	Folmer et al. (1994)
HCO2198 (antisense)	TAAACTTCAGGGTGACCAAAAAATCA			

References

- Braig HR, Zhou W, Dobson SL and O'Neill SL (1998) Cloning and characterization of a gene encoding the major surface protein of the bacterial endosymbiont Wolbachia pipientis. J. Bacteriol. 180: 2373–2378.
- Caspi-Fluger A, Inbar M, Mozes-Daube N, Mouton L, Hunter MS and Zchori-Fein E (2011) Rickettsia 'in' and 'out': two different localization patterns of a bacterial symbiont in the same insect species. PLoS One 6:e21096.
- Folmer O, Black M, Hoeh W, Lutz R and Vrigenhoek R (1994) DNA primers for amplification of mitochondrial cytochrome c oxidase subunit I from diverse metazoan invertebrates. Mol. Mar. Biol. Biotechnol. 3: 294-299.
- Fukatsu T and Nikoh N (1998) Two intracellular symbiotic bacteria of the mulberry psyllid Anomoneura mori (Insecta, Homoptera). Appl. Environ. Microbiol. 64:3599–3606.
- Gottlieb Y, Ghanim M, Chiel E, Gerling D, Portnoy V, Steinberg S, Tzuri G, Horowitz AR, Belausov E, Mozes-Daube N, Kontsedalov S, Gershon M, Gal S, Katzir N and Zchori-Fein E (2006) Identification and localization of a Rickettsia sp. in Bemisia tabaci (Homoptera : Aleyrodidae). Appl. Environ. Microbiol. 72: 3646–3652.
- Haselkorn TS, Markow TA, Moran NA (2009) Multiple introductions of the Spiroplasma bacterial endosymbiont into Drosophila. Molecular Ecology 18: 1294–1305.
- Montenegro H, Solferini VN, Klaczko LB and Hurst GD (2005) Male-killing Spiroplasma naturally infecting Drosophila melanogaster. Insect Mol. Biol. 14: 281–287.
- Simon C, Buckley TR, Frati F, Stewart JB and Beckenbach AT (2006) Incorporating molecular evolution into phylogenetic analysis, and a new compilation of conserved polymerase chain reaction primers for animal mitochondrial DNA. Annu. Rev. Ecol. Evol. Syst. 37: 545–579.
