# Supplementary material for: A Nightmare for Males? A Maternally Transmitted Male-Killing Bacterium and Strong Female Bias in a Green Lacewing Population
Source: PLoS One. 2016 Jun 15;11(6):e0155794. doi: 10.1371/journal.pone.0155794 (PMC4909225; doi:10.1371/journal.pone.0155794)
Supplement: S2 Table — (PDF) [file pone.0155794.s003.pdf]

S2 Table. Survivorship and sex ratio of F1 offspring.

	Individual no.	Infection status	Eggs laid	No. of died individuals (F1)				Egg hatch rate	Survival rate	Female	Male	Proportion of females (n)	Line code
				Eggs	Larvae	Pupae	Pharate-adult						
**(a) All-female**	#5	S+R-	156	85	ND	ND	ND	0.455	0.404	63	0	1.000 (63)***	MK5
	#10	S+R-	146	11	55	6	8	0.925	0.452	66	0	1.000 (66)***	MK10
	#27	S+R-	64	21	11	0	0	0.672	0.500	32	0	1.000 (32)***	MK27
	#28	S+R-	112	9	41	2	2	0.920	0.518	58	0	1.000 (58)***	MK28
	#34	S+R-	73	9	28	0	0	0.877	0.493	36	0	1.000 (36)***	MK34
	#48	S+R-	57	14	29	7	1	0.754	0.105	6	0	1.000 (6)*	MK48
	#3	S+R+	103	57	ND	ND	ND	0.447	0.359	37	0	1.000 (37)***	MK3
	#7	S+R+	130	40	30	0	0	0.692	0.462	60	0	1.000 (60)***	MK7
	#8	S+R+	131	60	11	1	0	0.542	0.450	59	0	1.000 (59)***	MK8
	#12	S+R+	104	32	29	0	0	0.692	0.413	43	0	1.000 (43)***	MK12
	#15	S+R+	121	51	17	0	10	0.579	0.355	43	0	1.000 (43)***	MK15
	#17	S+R+	129	25	54	1	3	0.806	0.357	46	0	1.000 (46)***	MK17
	#19	S+R+	101	47	7	0	1	0.535	0.455	46	0	1.000 (46)***	MK19
	#20	S+R+	89	53	4	0	0	0.404	0.360	32	0	1.000 (32)***	MK20
	#22	S+R+	83	25	17	2	0	0.699	0.470	39	0	1.000 (39)***	MK22
	#26	S+R+	154	9	20	55	0	0.942	0.455	70	0	1.000 (70)***	MK26
	#29	S+R+	111	21	36	1	1	0.811	0.468	52	0	1.000 (52)***	MK29
	#32	S+R+	169	50	54	0	4	0.704	0.361	61	0	1.000 (61)***	MK32
	#33	S+R+	66	27	4	0	0	0.591	0.530	35	0	1.000 (35)***	MK33
	#43	S+R+	91	44	4	0	1	0.516	0.462	42	0	1.000 (42)***	MK43
	#45	S+R+	61	37	9	0	1	0.393	0.230	14	0	1.000 (14)**	MK45
**(b) Female-biased sex ratio**	#9	S+R+	140	17	39	24	4	0.879	0.400	49	7	0.875 (56)***	MK'9
	#30	S+R+	105	16	18	3	0	0.848	0.648	46	22	0.676 (68)*	MK'30
	#2	S-R+	189	91	ND	ND	ND	0.519	0.434	70	12	0.854 (82)***	MK'2
**(c) Normal sex ratio**	#21	S+R+	166	9	6	0	4	0.946	0.916	72	80	0.474 (152)	N21
	#46	S+R+	60	1	17	6	4	0.983	0.533	14	18	0.438 (32)	N46
	#4	S-R+	190	18	ND	ND	ND	0.905	0.763	72	73	0.497 (145)	N4
	#18	S-R+	69	5	1	1	5	0.928	0.826	30	27	0.526 (57)	N18
	#24	S-R+	96	11	13	1	6	0.885	0.677	43	22	0.661 (65)	N24
	#31	S-R+	84	8	4	0	4	0.905	0.810	40	28	0.588 (68)	N31
	#44	S-R+	122	16	6	1	7	0.869	0.754	49	43	0.533 (92)	N44
	#6	S-R-	166	21	ND	ND	ND	0.873	0.657	51	58	0.468 (109)	N6
	#13	S-R-	90	3	5	0	9	0.967	0.811	30	43	0.411 (73)	N13
	#14	S-R-	118	46	6	1	8	0.610	0.483	28	29	0.491 (57)	N14
	#1	NA	172	42	ND	ND	ND	0.756	0.552	41	54	0.432 (95)	N1
Total										1575	516	0.826 (2091)	

*P < 0.05; ***P < 0.001

S+ and S- indicate positive and negative for Spiroplasma, respectively.

R+ and R- indicate positive and negative for Rickettsia, respectively.

ND, no data
