# Supplementary material for: A Nightmare for Males? A Maternally Transmitted Male-Killing Bacterium and Strong Female Bias in a Green Lacewing Population
Source: PLoS One. 2016 Jun 15;11(6):e0155794. doi: 10.1371/journal.pone.0155794 (PMC4909225; doi:10.1371/journal.pone.0155794)
Supplement: S3 Table — (PDF) [file pone.0155794.s004.pdf]

S3 Table. qPCR data performed for wild-caught females, which were DNA extracted posterior to oviposition.

Samples	Infection status	F1 sex ratio	Mean conc. (copies/ul)			gltA/COII (Ric)	spoT/COII (Spiro)	gltA/spoT (Ric/Spiro)	Egg hatch rate
			COII	gltA (Ric)	spoT (Spiro)				
#5	S+R-	all-female	2.63E+06	-	1.19E+02	-	4.53E-05	-	0.455
#10	S+R-	all-female	1.88E+06	-	1.71E+01	-	9.10E-06	-	0.925
#27	S+R-	all-female	2.06E+06	-	8.31E+01	-	4.04E-05	-	0.672
#28	S+R-	all-female	2.03E+06	-	1.68E+02	-	8.26E-05	-	0.92
#34	S+R-	all-female	2.86E+06	-	1.98E+02	-	6.92E-05	-	0.877
#48	S+R-	all-female	3.41E+06	-	2.00E+02	-	5.87E-05	-	0.754
#3	S+R+	all-female	3.75E+06	5.70E+04	1.32E+02	1.52E-02	3.53E-05	430.47	0.447
#7	S+R+	all-female	2.37E+06	7.97E+04	5.80E+01	3.37E-02	2.45E-05	1373.18	0.692
#8	S+R+	all-female	2.07E+06	2.42E+04	9.32E+01	1.17E-02	4.49E-05	260.23	0.542
#12	S+R+	all-female	1.96E+06	8.51E+04	3.15E+02	4.34E-02	1.60E-04	270.43	0.692
#15	S+R+	all-female	1.67E+06	3.22E+04	9.79E+01	1.92E-02	5.85E-05	328.86	0.579
#17	S+R+	all-female	1.35E+06	5.31E+04	1.95E+02	3.93E-02	1.44E-04	272.13	0.806
#19	S+R+	all-female	2.92E+06	3.55E+04	1.26E+02	1.22E-02	4.32E-05	281.44	0.535
#20	S+R+	all-female	2.03E+06	3.41E+04	1.13E+01	1.68E-02	5.59E-06	3011.32	0.404
#22	S+R+	all-female	3.63E+06	1.44E+04	1.97E+02	3.97E-03	5.44E-05	72.86	0.699
#26	S+R+	all-female	3.56E+06	6.19E+04	7.93E+01	1.74E-02	2.23E-05	780.44	0.942
#29	S+R+	all-female	3.68E+06	1.41E+05	3.13E+02	3.83E-02	8.49E-05	451.53	0.811
#32	S+R+	all-female	4.34E+06	3.62E+05	4.35E+02	8.33E-02	1.00E-04	832.66	0.704
#33	S+R+	all-female	3.06E+06	7.66E+04	2.83E+02	2.50E-02	9.26E-05	270.09	0.591
#43	S+R+	all-female	3.09E+06	1.26E+04	3.08E+02	4.09E-03	9.97E-05	40.98	0.516
#45	S+R+	all-female	4.93E+06	3.86E+04	6.66E+02	7.82E-03	1.35E-04	57.93	0.393
#9	S+R+	female-biased	2.26E+06	6.82E+04	6.50E+01	3.02E-02	2.87E-05	1049.05	0.879
#30	S+R+	female-biased	3.65E+06	1.21E+05	3.89E+02	3.31E-02	1.07E-04	310.26	0.848
#2	S-R+	female-biased	1.49E+06	5.31E+04	-	3.56E-02	-	-	0.519
#21	S+R+	normal	1.70E+06	3.61E+04	3.12E+01	2.13E-02	1.84E-05	1154.23	0.946
#46	S+R+	normal	1.76E+06	6.04E+04	2.13E+01	3.43E-02	1.21E-05	2828.93	0.983
#4	S-R+	normal	3.33E+06	7.88E+04	-	2.37E-02	-	-	0.905
#18	S-R+	normal	2.16E+06	2.31E+04	-	1.07E-02	-	-	0.928
#24	S-R+	normal	2.68E+06	3.03E+04	-	1.13E-02	-	-	0.885
#31	S-R+	normal	2.29E+06	4.67E+04	-	2.04E-02	-	-	0.905
#44	S-R+	normal	3.17E+06	7.59E+04	-	2.40E-02	-	-	0.869
#6	S-R-	normal	2.26E+06	-	-	-	-	-	0.873
#13	S-R-	normal	1.21E+06	-	-	-	-	-	0.967
#14	NA	normal	1.92E+06	-	-	-	-	-	0.61

S+ and S- indicate positive and negative for Spiroplasma, respectively.
R+ and R- indicate positive and negative for Rickettsia, respectively.
NA: not examined
