# Supplementary material for: A Nightmare for Males? A Maternally Transmitted Male-Killing Bacterium and Strong Female Bias in a Green Lacewing Population
Source: PLoS One. 2016 Jun 15;11(6):e0155794. doi: 10.1371/journal.pone.0155794 (PMC4909225; doi:10.1371/journal.pone.0155794)
Supplement: S4 Table — (PDF) [file pone.0155794.s005.pdf]

S4 Table. Bacterial contents inferred from the 16S rRNA gene sequencing.

Wild-caught females (mothers)	Results of diagnostic PCR	No. of inserted colonies[1]			
		Rickettsia	Spiroplasma	Others	Total
#2	S-R+	24	0	-	24
#3	S+R+	12	2	1 (Asaia sp.)	15
#5	S+R-	0	25	-	25
#9	S+R+	11	2	-	13
#20	S+R+	22	2	1 (Acinetobacter sp.)	25

[1]Results of sequencing
