# Supplementary material for: A Nightmare for Males? A Maternally Transmitted Male-Killing Bacterium and Strong Female Bias in a Green Lacewing Population
Source: PLoS One. 2016 Jun 15;11(6):e0155794. doi: 10.1371/journal.pone.0155794 (PMC4909225; doi:10.1371/journal.pone.0155794)
Supplement: S5 Table — (PDF) [file pone.0155794.s006.pdf]

S5 Table. Infection status of F1 offspring.

Line	Sex	Infection status					Total
		S+R+	S+R-	S-R+	S-R-	NA	
**(a) Doubly infected lines**							
MK3(S+R+)	Female	8	0	0	0	29	37
MK7(S+R+)	Female	8	0	0	0	52	60
MK8(S+R+)	Female	8	0	0	0	51	59
MK12(S+R+)	Female	8	0	0	0	35	43
MK20(S+R+)	Female	8	0	0	0	24	32
MK26(S+R+)	Female	8	0	0	0	62	70
MK43(S+R+)	Female	8	0	0	0	34	42
MK'9(S+R+)	Female	12	0	0	0	37	49
	Male	0	0	7	0	0	7
MK'30(S+R+)	Female	11	1	0	0	34	46
	Male	12	0	0	0	10	22
N21(S+R+)	Female	12	0	0	0	60	72
	Male	11	0	1	0	68	80
N46(S+R+)	Female	9	0	0	0	5	14
	Male	12	0	0	0	6	18
**(b) Rickettsia-infected line**							
MK'2(S-R+)	Female	0	0	8	0	62	70
	Male	0	0	8	0	4	12

S+ and S- indicate positive and negative for Spiroplasma, respectively.
R+ and R- indicate positive and negative for Rickettsia, respectively.
NA, not analysed
